# Supplementary material for: Learning curve and surgical outcome of robotic assisted colorectal surgery with ERAS program
Source: Sci Rep. 2022 Nov 29;12:20566. doi: 10.1038/s41598-022-24665-w (PMC9709162; doi:10.1038/s41598-022-24665-w)
Supplement: Supplementary file 2 — Supplementary Information 2. [file 41598_2022_24665_MOESM2_ESM.zip › RAL Raw data-Ñ[▒K/083/OP N_ñw╝╨░O▒Kñσ.pdf]

W52 / W52

臺中榮民總醫院
手術室護理記錄

[redacted]

基本資料

- 室別： 開刀房-17室，手術科別： CRS，手術類別： 常規，日期： 2021/05/14
- 進入時間： 1240 ； 麻醉開始時間： 1255 ； Time Out時間： 1350 ； 劃刀時間： 1351 ； 手術結束時間： 1950 送出時間： 2010 ；
- 麻醉方式： GE
  診斷：Malignant tumor of rectum
  手術名稱：Robot Assisted Low Anterior Resection
  手術局部用藥：NIL.
  術前預防性抗生素：
  - 抗生素名稱一： Veterin 1GM IVD由麻醉護理師給予
  - 時間： 1345
  - 上次簽章: 張秀敏

  ，追加預防性抗生素：
  - 抗生素名稱一： Veterin 1GM IVD由麻醉護理師給予
  - 時間： 1745
  - 上次簽章: 陳心慈
- 手術醫師： 蔣鋒帆，楊曙榮，古朋育，陳瑨夫
- 麻醉醫師： VS 蔡佩馨
-
  - 刷手護士A ： 吳婉寧／陳盈妤，時間： 1240，接替刷手一： 呂昭蓉，時間： 1500，接替刷手二： 張秀敏，時間： 1730
  - 流動護士A ： 呂昭蓉／張秀敏，時間： 1240，接替流動一： 陳心慈，時間： 1700，接替流動二： 呂昭蓉，時間： 1800

皮膚狀況

A-V Shunt： 無
術前： 完整
記錄者： 上次簽章: 張秀敏
術後： 完整
記錄者： 呂昭蓉

手術準備

手術臥式 單一手術臥式

-
  - 手術臥式： 截石臥式
  - 使用附件： 溫毯 38 ℃，腳架
  - 皮膚準備： 10%Aq Betadine，Hibiscrub，2% Chlorhexidine
  - 電燒： 使用電燒: 電極片位置： 右大腿，記錄者： 上次簽章: 張秀敏

止血帶T： 無

植入物

- 植入物名稱/號碼/數量/部位：coviden GIA 80-3.8mm*1
  ENDO GIA 45-43.mm*3/rectum

，記錄者： 上次簽章: 張秀敏

標本送檢

- 細菌培養 無
- 細胞學 無
- 冰凍切片 無
- 病理標本
  組織名稱一：rectum
  ，

組織名稱二： IMA root
,
組織名稱三： distal cut end

- 截肢 無

標本交付 標本： 1 袋 ， 標本： 0 盒 ， 標本： 2 瓶 ， 細胞學： 0 管 ， Culture Tube： 0 管 ， 被交付醫師： R 古朋育 ，

- 記錄者： 呂昭蓉
- 時間： 2010

傷口術後敷料

傷口敷料： 傷口敷料： 無

- 請於圖上標示部位：

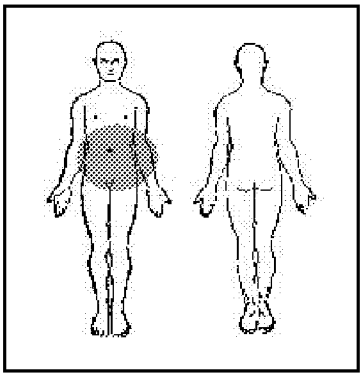

- 傷口敷料： 紗布

導管

導管	天數	導管資訊	插入日期	插入時間	更換日期	拔除日期	拔除時間	拔除原因	預計拔除/更換	預計拔除/更換日期
Epidural PCA(1)	1	部位:硬脊膜;	2021/05/14	1230						
Foley(1)	1	號碼:16 Fr.; 部位:膀胱;	2021/05/14	1322						
Double Lumen CVC(1)	1	號碼:8 Fr.; 深度:15cm; 部位:jugular (右); 象限:RU;	2021/05/14	1320						
A-line (1)	1	號碼:20 G; 部位:左手;	2021/05/14	1315						

用物計數

- 手術中出血量： minimal c.c.
- 手術中尿量： 少 c.c.
-
  - 其它： 無

用物計數： 單一切口

- 1.器械： 正確 ，2.縫針： 正確 ，3.刀片 ： 正確 ，4.紗布： 正確 ，5.腹部墊： 未使用 ，6.紗球： 未使用 ，7.PEANUTS ： 未使用 ，8.紗條： 未使用 ，9.絲帶： 正確 ，10.針頭： 未使用 ，11.Vessel loop： 未使用 ，12.Boots： 未使用 ，13.Jelco： 未使用 ，14.棉條： 未使用 ，
  - 第一次：
    - 刷手護士 ： 張秀敏
    - 流動護士： 呂昭蓉
    - 時間： 1850

,
  - 第二次：
    - 刷手護士 ： 張秀敏
    - 流動護士： 呂昭蓉
    - 時間： 1855

,
  - 第三次：
    - 刷手護士 ： 張秀敏
    - 流動護士： 呂昭蓉

- 時間： 1900

備註

備註： 達文西30度鏡No.1

- 記錄者： 上次簽章: 張秀敏
- 時間： 1300

填寫護理人員 呂昭蓉

正式摘要

		W52 / W52			

doctorRank:B
